# Supplementary material for: Rapid shifting of a deep magmatic source at Fagradalsfjall volcano, Iceland
Source: Nature. 2022 Sep 14;609(7927):529–34. doi: 10.1038/s41586-022-04981-x (PMC9477742; doi:10.1038/s41586-022-04981-x)
Supplement: Supplementary file 1 — Supplementary Sections 1–6; legends for the Supplementary tables and References. [file 41586_2022_4981_MOESM1_ESM.pdf]

nature portfolio

https://doi.org/10.1038/s41586-022-04981-x

## Supplementary information

# Rapid shifting of a deep magmatic source at Fagradalsfjall volcano, Iceland

In the format provided by the authors and unedited

**Supplementary information – additional information not provided in the manuscript text and/or methods**


S1. Sampling and sample description

S2. Petrography

S3. Mineral chemistry

S4. Glass and melt inclusion chemistry

S5. Analytical Details: Whole rock trace element and isotope analysis

S6. Analytical Details: Major volatiles and trace element analysis in melt inclusions


**Overview of Supplementary Tables (ST) – see Excel file**


ST1. Sample overview

ST2. Whole rock ICP-OES, major element data

ST3. Whole rock and groundmass glass ICP-MS, Trace element data

ST4. Whole rock and groundmass glass, Isotope data

ST5. Clinopyroxene EPMA and P-T data

ST6. Olivine EPMA data

ST7. Plagioclase EPMA data

ST8: Spinel EPMA data

ST9: Groundmass glass EMPA and P-T data

ST10: Melt inclusion major element and P-T data

ST11: Melt inclusions, trace elements and volatiles

ST12: Volcanic gas chemistry

**S1. Sampling and sample description (Supplementary Table 1):** The 25 samples include fallout pumice-like tephra collected either directly upon deposition or shortly thereafter, incandescent semi-liquid and liquid lava quenched in water, as well as solidified lava samples (**Supplementary Table 1**). Unsolidified, low-viscosity pāhoehoe flows were sampled with a 2 m metal spoon and immediately quenched in tap water. Similarly, high-viscosity or simply incandescent ʻaʻā lavas were sampled and quenched in tap water. The ʻaʻā lavas are characterized by a less glassy structure and contained more crystalline groundmass than pāhoehoe flow samples. As the ʻaʻā lavas were at the surface for unknown periods of time between leaving the crater and sampling, these samples were given emplacement time ranges corresponding to known lava outbreaks associated with the sampled flow. Slabs of glassy naturally-cooled pāhoehoe lavas were collected when the emplacement date or range was known, thus filling in temporal gaps between quenched lava samples.

**S2. Petrography** (**Extended Data Figure 1**): Petrographic observations suggest that the Fagradalsfjall tephra and quenched lava samples contain minerals in three dominant grain sizes, (i) larger macrocrysts (from 0.5x1 mm in length) (ii) microphenocrysts (max 500 and 100 μm), and (iii) small microlites (below 50 μm in length).

Both microphenocrysts and the macrocrysts with more evolved compositions (see mineral chemistry below) show glomerophyric intergrowths between clinopyroxene, plagioclase and olivine (**Extended Data Figure 1**). The more primitive group of macrocrysts occasionally contain Cr-spinel inclusions (**Extended Data Figure 1**). Cr-spinel occurs also as a minor phase of the macrocryst cargo, although with smaller size (up to 0.5 mm). Macrocrysts are euhedral or subhedral, whereas microphenocrysts are euhedral. Most macrocrysts are chemically zoned. Olivines and plagioclases are most commonly normally zoned, but complex (primitive core, more evolved mantle, and then more primitive rim) zoning also occurs. Some clinopyroxene macrocrysts have a complex internal structure (e.g. sector zoning), others are homogeneous in the core, with or without a thin normally zoned rim. Plagioclase microphenocrysts are homogeneous, but olivine and clinopyroxene microphenocrysts are zoned (**Extended Data Figure 3**).

All macrocryst phases contain silicate melt inclusions, sometimes both in their cores and rims (**Extended Data Figure 1**). Olivine and clinopyroxene macrocryst cores have individual, spherical, glassy melt inclusions with size up to 100 - 150 μm in diameters. Melt inclusions in macrocryst rims are generally smaller, and sometimes they have irregular shapes. Plagioclase macrocryst cores commonly show sieve texture with randomly distributed spherical or elongated (negative crystal shaped) melt inclusions, which are occasionally >150 μm in diameter, but generally smaller (**Extended Data Figure 1)**. Spinel-hosted melt inclusions show similar features. The largest melt inclusions in each macrocryst type have a vapour bubble, which contains low-density $CO_2$, identified by Raman spectroscopy (not discussed in this paper). The smaller melt inclusions did not develop $CO_2$ bubbles.

**S3. Mineral chemistry (Supplementary Tables 6-8):** Variation of mineral compositions is shown in **Extended Data Figure 3.** Olivine compositions vary between forsterite content (Fo; calculated as Mg/[Mg+$Fe^{2+}$] mol%)of 80.6 to 90. Fo correlates positively with NiO (0.14-0.31 wt%). There is a significant overlap between core and rim compositions with two main olivine populations with $Fo_{88}$ and $Fo_{85}$ (**Extended Data Figure 3a**). Although there is a continuous compositional variation, we observe two main populations of olivine core compositions, primitive cores with the most common composition of $Fo_{88}$ and more evolved cores and rims on the primitive cores with $Fo_{85}$ (**Extended Data Figure 3a**). Moreover, we observe zonation in microphenocrysts with more evolved rims down to $Fo_{80}$. Assuming a $K_{DFeMg}^{Ol\text{-}liquid}$ of 0.3±0.03 of Roedder and Emslie (1970), $Fo_{85}$ core and rim compositions are close to equilibrium with the carrier liquid, assumed to be represented by the tephra glass compositions, whereas $Fo_{88}$ cores are in chemical equilibrium with the primitive melt inclusions. Lastly, the rims with $Fo_{80}$ are in equilibrium with groundmass glass compositions measured in some high-crystallinity quenched lava samples.

Clinopyroxene core and rim compositions overlap similarly, as observed in olivines. There is an overall positive correlation between Mg# (calculated as Mg/[Mg+$Fe^{2+}$] mol%) and the $Al_2O_3/TiO_2$ ratio in all clinopyroxenes, with a larger spread in these ratios towards more primitive compositions. Based on the $KD_{FeMg}^{cpx\text{-}liquid}$ of 0.27±0.03 equilibrium criteria of Putirka et al. (2008) and a $Fe^{2+}/Fe^{3+}$ = 0.9, the carrier liquid is in equilibrium with the cpx Mg#84-85, whereas the most primitive core compositions are in Fe-Mg equilibrium with the whole rock compositions and moderately primitive melt inclusions. Clinopyroxenes in equilibrium with the most primitive melt inclusion compositions were not found. Note that Fe-Mg equilibrium in itself does not necessarily mean equilibrium between these melt and clinopyroxene compositions (see other equilibrium criteria in Methods).

In contrast with clinopyroxenes and olivines, plagioclases cover two distinct compositional ranges with a compositional gap. The more primitive core composition group has anorthite content (An; calculated as An = Ca/[Ca+Na+K] mol%) of 84 to 91, whereas the more evolved one has An between 76 to 79 (**Extended Data Figure 3c**). The second group overlaps in composition with the rims of the macrocrysts with the more primitive cores. While plagioclase cores might be in equilibrium with primitive melt inclusions (based on the equilibrium criteria of Namur et al. 2011), some rims show equilibrium with groundmass glass or whole rock compositions. Many rim and microphenocryst compositions are, however, too evolved to be in equilibrium with the groundmass glass.

Spinels have highly variable Mg# and Cr# (calculated as Cr/[Cr+Al+$Fe^{3+}$] mol%) between 55-65 and 38-50, respectively. Core compositions on average are more Cr-rich than rims. However, core and rim compositions overlap. Based on Cr# values, we distinguish 3 main populations. The population with the most common values of 46-48 is typical for spinel cores. The second population (typically Cr# of 42-44) occurs both in cores and rims, whereas the population with the lowest values (most typically ~40) are common in spinel rims.

In summary, some of the macrocrysts are too primitive to have crystallized from the Fagradalsfjall carrier liquid and are likely to represent xenocrysts and/or antecrysts from crystal mush horizons at depth (see main text for further interpretations). In contrast, macrocryst rims and microphenocrysts have more evolved compositions and are close to the predicted equilibrium with the groundmass glass, consistent with their crystallization in the crustal conduit during ascent and within the lava flow.

**S4. Glass and melt inclusion chemistry (Supplementary Tables 9 and 10):** Groundmass glass of quenched lava samples have MgO content between 6.7 and 8.0 wt% (**Extended Data Figure 2a**), with the lower values in the higher crystallinity samples. The composition of glass in naturally quenched tephra overlaps with those in the quenched lava at the high MgO contents (MgO between 7.1 and 9.0 wt%). Some of the most evolved melt inclusions trapped in olivine rims overlap with groundmass glass compositions. $TiO_2$ content of groundmass glasses and evolved melt inclusions is between 1.0 and 1.2 wt%, with higher values in the high-crystallinity glass samples. $TiO_2$ correlates negatively with MgO. $K_2O$ content in groundmass glasses varies between 0.10 and 0.37 wt%. Although $K_2O$ contents correlate broadly negatively with MgO, there is a distinct group of the groundmass glass composition with elevated $K_2O$ relative to other glasses. The sample with these compositions was collected on the 25$^{th}$ day of the eruption. Compared to groundmass glass compositions, whole rock samples have higher MgO (8.8-10.0 wt%) and lower $TiO_2$ contents (0.95-1.12).

Silicate melt inclusions cover a very large compositional range, with MgO content in PEP-corrected melt inclusions between 7.6 and 12.3 wt% (**Extended Data Figure 2c**). In the case of olivine-hosted melt inclusions, two groups can be distinguished. The composition of the more evolved group generally falls between the groundmass glass and whole rock compositions (overlapping with both), whereas the more primitive group has MgO contents over 9.7 wt%. In fact, the most primitive melt inclusions are trapped in primitive olivine cores. Among the most primitive melt inclusions, a $K_2O$-rich (0.25-0.45 wt%) and a $K_2O$-poor (below 0.05 wt%) group can be distinguished. The $K_2O$-rich group also has distinctly higher FeO contents at a given MgO compared to those in the $K_2O$-poor group. The $K_2O$-poor primitive melt inclusions dominate the samples. Plagioclase-hosted melt inclusions have MgO contents between 8.5 and 10.0 wt% and they broadly overlap with whole rock compositions, though covering a larger range between the more primitive olivine-hosted group and the groundmass glass. Clinopyroxene-hosted melt inclusions overlap in composition with the plagioclase-hosted ones.

**S5. Analytical Details: Whole rock trace element and isotope analysis**

**ICP-MS:** Glass fragments (0.5 – 1 mm) were picked from crushed portions of lavas for trace element analysis. Whole rock powders were prepared by hand-grinding whole lava fragments with an agate mortar and pestle. All further sample processing was performed in a metal-free clean laboratory. All acids used were double distilled, and all water prepared by distillation in

a quartz still, subsequently purified to 18.2MΩ resistivity using a MembraPure Astacus. Glass fragments were rinsed in water, while whole rock powers were not rinsed. Samples were digested in an HF-$HNO_3$ mixture. The samples were converted to nitrate form and dissolved in 2N $HNO_3$. An aliquot of each digested sample was transferred to an acid-leached 10 mL vial, spiked with an In-Re internal standard, and made up to volume with 2% $HNO_3$ for a dilution factor of ~5000. This solution was analyzed for trace elements (see table for elements analysed) on a ThermoFisher iCap RQ Quadrupole Inductively-coupled Plasma Mass Spectrometer (ICP-MS) at the Institute of Earth Sciences, University of Iceland with a quartz inlet system. Data reduction included corrections for matrix suppression, drift in instrument sensitivity, reagent blanks, and interferences by oxides, doubly-charged ions and isobaric overlaps. The data were calibrated with a suite of international rock standard reference materials (BIR-1, BHVO-2, BCR-2, AGV-2), using the preferred values from the GeoRem database (http://georem.mpch-mainz.gwdg.de). Standards were chosen to represent material close in composition to the samples (W-2), an enriched (BHVO-2) standard and a depleted (BIR-1) to assess accuracy. Precision was assessed from replicate digestions of the W-2 rock standard. Replicate digestions of two samples were used to assess data quality (all ICP-MS data, including standard replicates, are reported in Supplementary Table 3).

**MC-ICP-MS:** Radiogenic isotopes were analyzed at the Institute of Earth Sciences, University of Iceland using methods similar to those described by Halldórsson et al. (2018). Hand-picked glass chips from field-quenched lavas were dissolved in a metal-free clean laboratory environment, leached once with 6 M HCl and rinsed twice with MQ-water in a hot (~50°C) ultrasonic bath. Digestion of whole-rock and glass samples was made in an HF:$HNO_3$ mixture. Ion chromatography for Pb separation was based on the "HCl-method" of Kamber and Gladu (2009). The extracted Pb was converted to nitrate form and dissolved in 2% $HNO_3$ for analysis. The Pb isotopic compositions of samples were measured using a Nu Plasma multi-collector inductively-coupled plasma mass-spectrometer (MC-ICP-MS). Samples were introduced into the plasma using an Apex-Q quartz nebulizer. All measurements were made in static mode and reproducibly of all internal standards (SRM-981) obtained over the analytical period is reported in Supplementary Table 4. For Pb isotopic analyses samples were spiked each session with fresh Tl solution as an internal standard at a Pb/Tl ratio of 2-4. $^{205}Tl/^{203}Tl$ spike (SRM-997) for correction of instrumental mass fractionation assuming an exponential law and a constant $^{205}Tl/^{203}Tl$ ratio of 2.3889[35]. The Hg beam was monitored at mass 202, and a correction for interference of $^{204}Hg$ with $^{204}Pb$ made using natural $^{204}Hg/^{202}Hg$ = 0.229 corrected for mass fractionation. Internal reproducibility of the Pb-isotopic ratios was based on multiple analyses of the SRM-981 Pb reference material. During each run SRM-981 was run as a bracketing standard and all Pb-isotopic ratios are normalized to the SRM-981 values of Baker et al. (2004): $^{206}Pb/^{204}Pb$ = 16.9416, $^{207}Pb/^{204}Pb$ = 15.4998, and $^{208}Pb/^{204}Pb$ = 36.7249. External reproducibility is based on multiple analyses of reference material (USGS AGV-2, BCR-2) (Supplementary Table 4). The values obtained during this analytical session are in good agreement with published values, indicating good accuracy of analysis. Procedural blanks

were considered negligible relative to the amount of Pb extracted and therefore no blank correction was performed.

**S6. Analytical Details: Major volatiles and trace element analysis in melt inclusions**

**SIMS**: The trace elements Ni, Sr, Y, Zr, Nb, La, Ce, Nd, Sm, Eu, Gd, Dy, Er, and Yb in olivine and plagioclase hosted melt inclusions and matrix glass were measured using a CAMECA IMS1280 SIMS instrument at the NordSIM facility, Swedish Museum of Natural History, Stockholm. We followed the same analytical procedure as described in Caracciolo et al. (2022).

The contents of $H_2O$ and $CO_2$ were determined in melt inclusions and matrix glass using the same SIMS instrument used for trace elements. Prior to the analytical session, the two epoxy sample mounts and the reference materials mount were continuously pumped in the multi-sample customized sample exchange airlock at <3 x $10^{-8}$ torr for four days to minimize volatile background from outgassing. The liquid $N_2$ cold trap on the sample chamber was used to achieve a pressure of ca. 3 x $10^{-9}$ torr during analysis. The Hyperion H201 RF Plasma generated a critically focused $O_2^-$ primary beam, which was tuned to 2 nA and rastered over an area of 10 x 10 µm during analysis. Prior to data acquisition, the gold coating and potential sub-surface contamination was removed using a 120 sec. pre-sputter with a 10 nA primary beam over a 20 x 20 µm rastered area. Sputtered secondary ions were admitted to the mass spectrometer using high transmission lens settings (transfer magnification = 160x), with the entrance slit set to 75 µm in order to retain a wide flat-topped peak in the 600 µm exit slit (corresponding to a nominal mass resolution of 2000, M/ΔM). The field aperture was set to give a field of view on the sample of 15 x 15 µm, marginally larger than the analysed spot but smaller than the pre-sputtered area in order to avoid sampling of contaminated surface by any beam aberrations; for the same reason, the Dynamic Transfer Optical System (DTOS), which deflects secondary ions from rastered areas back onto the ion optic axis, was not utilized. Following the pre-sputter, the secondary ions were automatically centered in the field aperture and the sample high voltage adjusted to yield the maximum in the 45 eV energy window, both using the matrix species $^{28}Si^{2+}$ (nominal mass 14). The mass calibration was corrected for any drift by scanning the secondary magnet field for the species $^{1}H^{+}$ and $^{28}Si^{2+}$. Because of its low mass, the trajectory of the $^{1}H^{+}$ ions through the instrument is strongly influenced by stray magnetic fields and so the tuning was adjusted for this species using secondary ion tuning devices to maintain the same peak shape as the higher mass species; these specific settings were coded into the analysis routine using Optional Instrument Parameters (OIPs) in the CAMECA CIPS software. Each analysis comprised eight peak hopping scans through the species $^{1}H^{+}$, $^{12}C^{+}$ and $^{28}Si^{2+}$, with integration times in the ion counting electron multiplier of 4 seconds/species/cycle; the OIP was applied to $^{1}H^{+}$ at each cycle (and during mass calibration adjustment).

Conversion of $^{1}H^{+}/^{28}Si^{2+}$ and $^{12}C^{+}/^{28}Si^{2+}$ ratios to $H_2O$ and $CO_2$ was achieved using a set of volatile doped basaltic glasses of Shishkina et al. (2010) (kindly provided by R. Almeev, Univ. of Hannover) including M5 (0.64 wt% $H_2O$, 990 ppm $CO_2$), M15 (1.54 wt%, 60 ppm), M43 (2.662%, 3172 ppm), M60 (5.89%, 5952 ppm, R. Almeev pers. comm.) and the nominally

volatile-free glass N72. The $M^+/Si^{2+}$ calibration curves were fitted as second order polynomials through the origin and all four M-series glasses for $H_2O$, omitting M60 for $CO_2$. The average $M^+/Si^{2+}$ ratios obtained from N72 using these calibration curves corresponded to an effective background of 0.035 wt % $H_2O$ and 4 ppm $CO_2$, which were subtracted from the determined sample concentrations. Both sample mounts also included Icelandic glass A35 from which an $H_2O$ content of 0.32 wt % (Halldórsson et al. 2016) and $CO_2$ content of 44 ppm (S.A. Halldórssson, unpublished data), both SIMS determinations made at the Department of Terrestrial Magnetism, Carnegie Institution of Washington using the methods of Hauri et al. (2002). For one mount, the A35 values reproduced the DTM numbers accurately, but for the second mount, which had poorly cured epoxy, the A35 analyses revealed an excess background corresponding to 0.024 wt% $H_2O$ and 54 ppm $CO_2$, which was applied to the unknown targets in that mount.

**References:**

Baker, J., Peate, D., Waight, T. & Meyzen, C. Pb isotopic analysis of standards and samples using a 207Pb-204Pb double spike and thallium to correct for mass bias with a double-focusing MC-ICP-MS. *Chemical Geology* **211**, 275–303 (2004).

Caracciolo, A., Halldórsson, S.A., Bali, E., Marshall, E.W., Jeon, H., Whitehouse, M.J., Barnes, J.D., Gudfinnsson, G.H., Kahl, M., Hartley, M.E., 2022. Oxygen isotope evidence for progressively assimilating trans-crustal magma plumbing systems in Iceland. *Geology*. doi.org/10.1130/G49874.1.

Halldórsson, S. A. *et al.* Subducted lithosphere controls halogen enrichments in the Iceland mantle plume source. *Geology* **44**, 679–682 (2016).

Halldórsson, S. A. *et al.* Petrology and geochemistry of the 2014–2015 Holuhraun eruption, central Iceland: compositional and mineralogical characteristics, temporal variability and magma storage. *Contributions to Mineralogy and Petrology* **173**, 1–25 (2018).

Hauri, E. *et al.* SIMS analysis of volatiles in silicate glasses 1. Calibration, matrix effects and comparisons with FTIR. *Chemical Geology* **183**, 99–114 (2002).

Jochum, K.P., Stoll, B., Herwig, K., Willbold, M., Hofmiann, A.W., Amini, M., Aarburg, S., Abouchami, W., Hellebrand, E., Mocek, B., Raczek, I., Stracke, A., Alard, O., Bouman, C., Becker, S., Dücking, M., Brätz, H., Klemd, R., De Bruin, D., Canil, D., Cornell, D., De Hoog, C.J., Dalpé, C., Danyushevshy, L., Eisenhauer, A., Gao, Y., Snow, J.E., Groschopf, N., Günther, D., Latkoczy, C., Guillong, M., Hauri, E.H., Höfer, H.E., Lahaye, Y., Horz, K., Jacob, D.E., Kasemann, S.A., Kent, A.J.R., Ludwig, T., Zack, T., Mason, P.R.D., Meixner, A., Rosner, M., Misawa, K., Nash, B.P., Pfänder, J., Premo, W.R., Sun, W.D., Tiepolo, M., Vannucci, R., Vennemann, T., Wayne, D., Woodhead, J.D., 2006. MPI-DING reference glasses for in situ microanalysis: New reference values for element concentrations and isotope ratios. *Geochemistry, Geophys. Geosystems* **7**. https://doi.org/10.1029/2005GC001060

Kamber, B. S. & Gladu, A. H. Comparison of Pb purification by anion-exchange resin methods and assessment of long-term reproducibility of Th/U/Pb ratio measurements by quadrupole ICP-MS. *Geostandards and Geoanalytical Research* **33**, 169–181 (2009).

Namur, O., Charlier, B., Toplis, M. J., & Vander Auwera, J. (2012). Prediction of plagioclase-melt equilibria in anhydrous silicate melts at 1-atm. *Contributions to Mineralogy and Petrology*, ***163***(1), 133-150.

Putirka, K. D. (2008). Thermometers and barometers for volcanic systems. *Reviews in mineralogy and geochemistry*, ***69***(1), 61-120.

Roeder, P. L., & Emslie, R. (1970). Olivine-liquid equilibrium. *Contributions to mineralogy and petrology*, ***29***(4), 275-289.

Shishkina, T. A., Botcharnikov, R. E., Holtz, F., Almeev, R. R. & Portnyagin, M. v. Solubility of H2O- and CO2-bearing fluids in tholeiitic basalts at pressures up to 500MPa. *Chemical Geology* **277**, 115–125 (2010).
